# Evaluation of MALDI-TOF for identification of *Vibrio cholerae* and *Vibrio parahaemolyticus* from growth on agar media

**DOI:** 10.1007/s00253-024-13385-y

**Published:** 2025-01-08

**Authors:** Swapan Banerjee, Annika Flint, Madeleine B. Brosseau, Kelly Weedmark, Bojan Shutinoski

**Affiliations:** 1 Vibrio Reference Laboratory, Bureau of Microbial Hazards, Health Canada, Ottawa, ON Canada; 2 Genomics Laboratory, Bureau of Microbial Hazards, Health Canada, Ottawa, ON Canada

**Keywords:** *Vibrio cholerae*, *Vibrio parahaemolyticus*, MALDI-TOF–MS, Identification

## Abstract

**Abstract:**

Two methods were compared for their ability to accurately identify *Vibrio* species of interest: whole genome sequencing as the reference method and MALDI-TOF MS (matrix-assisted laser desorption/ionization-time of flight mass spectrometry) proteome fingerprinting. The accuracy of mass spectrometry–based identification method was evaluated for its ability to accurately identify isolates of *Vibrio cholerae* and *Vibrio parahaemolyticus*. Identification result of each isolate obtained by mass spectrometry was compared to identification by whole genome sequencing (WGS). The MALDI-TOF MS system had excellent performance for identification of *V. cholerae* and *V. parahaemolyticus* isolates grown on a non-selective solid agar media. Unlike the biochemical characterization performed by API20E. In this study, 161 isolates (*V. cholerae, n* = 33; *V. parahaemolyticus, n* = 102; *V.* spp., *n* = 23; other enteropathogens, *Salmonella* and *E. coli*, *n* = 3) were used to assess accuracy. The MALDI-TOF MS system was able to accurately identify 100% (33/33) of the *V. cholerae* isolates and 99.9% (101/102) of *V. parahaemolyticus* isolates, with 100% for both sensitivity and specificity for *V. cholerae* and 99% sensitivity and 98% specificity for *V. parahaemolyticus*. Thus, mass spectrometry for bacterial identification is comparable to the WGS. Furthermore, in comparison to a biochemical characterization, the use of MALDI-TOF MS system shortens the analysis time from over 72 h to less than 24 h.

**Key points:**

*• V. cholerae and V. parahaemolyticus were successfully ID-ed by MALDI-TOF*

*• MALDI-TOF sensitivity and specificity parallels the WGS method of identification*

*• MALDI-TOF is several days faster than the battery of culture-dependent methods*

## Introduction

Consumption of raw or undercooked seafood is associated with infections caused by pathogenic *Vibrio* species. A key source of exposure to *Vibrio* spp. is the shellfish—predominantly the water-filtering mollusks (oysters, clams, mussels) that can concentrate bacteria found in the environment (Odeyemi 2016). There are 12 *Vibrio* species known to cause vibriosis, a human disease of the gastrointestinal tract (Baker-Austin et al. 2018). Vibriosis can be self limiting in healthy individuals, lasting up to several days. However, those with weakened immune system can develop severe illness that may be fatal. Of the pathogenic *Vibrio* species, *Vibrio parahaemolyticus* is most commonly associated with outbreaks (Yeung and Boor 2004), with *Vibrio vulnificus* having the highest morbidity rate (Johnston et al. 1985; Klontz et al. 1988).

Identification and characterization of foodborne bacteria by culture-based and biochemical techniques, such as nutrient utilization, can be advantageous due to their simplicity to perform and the requirement for basic laboratory equipment and have been used extensively for the classification of *Vibrio* spp. (Alsina and Blanch 1994; Noguerola and Blanch 2008). Biochemical characterization, in essence, relies on protein identification, one protein at a time, ascribing individual traits of the bacterial isolate of interest, for example, specific enzymatic activity as the ability to utilize various sugars as a sole source of energy: glucose, sucrose, arabinose, or urease activity or production of H_2_S, to name few. However, drawbacks of this approach include long turnaround time, up to several days are required to follow the complete microbiological identification scheme and high resource investment (e.g., hands on time and reagents) for each individual sample. Further, culture-based microbiological and biochemical techniques are often subjectively assessed by the operator (e.g., color development detected visually) and require experienced personnel familiar with the biology of *Vibrio* bacteria. Finally, some *Vibrio* species have the same or similar microbiological and biochemical features. This can prevent identification or result in misidentification of *Vibrio* species within the *Vibrio* clade (Kim et al. 2010; O'Hara et al. 2003). This study also confirmed that biochemical characterization as method for identification of *V. cholera* and *V. parahaemolyticus* is less efficient in comparison to mass spectrometry or whole genome sequencing. Hence, the need of identification methods based on biomarkers other than solely the biochemical traits of *Vibrio* species.

The advent of new technologies in food microbiology provides opportunities for faster identification and characterization of bacteria than was possible before. For example, the matrix-assisted laser desorption/ionization time-of-flight mass spectrometry system (MALDI-TOF MS) can rapidly and accurately identify to the genus and species level isolates of bacteria (Elbehiry et al. 2023; Erler et al. 2015; Faron et al. 2015; Li et al. 2022; Wang et al. 2023), yeast (Taverna et al. 2023), fungi (Moura-Mendes et al. 2023; Tome et al. 2019), and viruses (Hoyle and Downard 2023) by generating a characteristic mass spectra of charged proteins that are compared to a reference database. MALDI-TOF MS as a method for identification of purified bacteria relies on specific protein physical characteristics (i.e., mass and charge) that are unique to a species of interest. With a key distinction that the whole proteome or select portion of the proteome from a whole bacterial cell can be assessed simultaneously in generating species specific mass spectra as a unique biomarker to the species of interest.

Earlier attempts to profile *Vibrio* species by proteome analysis included 2D gel electrophoresis for mapping *Vibrio*’s proteome (Coelho et al. 2004; Kan et al. 2004; Zou et al. 2006). More recent studies have deployed MALDI-TOF mass spectrometry–based methods for identification of *Vibrio* by generating species‐identifying biomarker ions (Dieckmann et al. 2010; Hazen et al. 2009). However, commercial solutions for the use of MALDI-TOF mass spectrometry for species identification allow species identification from pure bacterial colonies within minutes. For example, the Biotyper™ system (Bruker Daltonics Inc.), a MALDI-TOF mass spectrometry–based technique for identification of bacterial isolates of interest. The original study by Faron et al. describing the Biotyper™ system was focused on clinical relevant species but did not incorporate *Vibrio* in the identification database (Faron et al. 2015). Since then, others report the use of commercially available MALDI-TOF with a focus on environmental species of the *Vibrio* genus, other than *V. cholerae* and *V. parahaemolyticus* (Moussa et al. 2021), and a study that report MALDI-TOF MS cluster assignment based on the species classification obtained by analysis of partial *rpoB* sequences of primarily environmental *Vibrio* strains (Erler et al. 2015). Although mass spectrometry–based methods can also misidentify some *Vibrio* species, they do offer greater resolution between species as they are based on proteome fingerprinting (Khot et al. 2012; Moussa et al. 2021).

Whole genome sequencing coupled with average nucleotide identity (Jain et al. 2018) is a high-resolution method for identification, characterization, and surveillance of any bacteria of interest Sundermann et al. (2022). However, if it is crucial to obtain identification results as fast as possible, WGS is not convenient for rapid identification, since the turnaround time for identification can be several days.

The objective of this study was to assess the suitability of the MALDI-TOF MS system for identification of seafood isolates of *V. cholerae* and *V. parahaemolyticus* grown directly on a non-selective solid agar media using WGS as a species classification method.

## Materials and methods

### Isolation of *Vibrio* species

Seafood samples from domestic or international origin were analyzed for the presence of *Vibrio* bacteria. Procedures described in ISO21872, BAM chapter 9 (Bacteriological Analytical Manual, 8th Edition, Revision A, 1998), and Banerjee and Farber (Banerjee and Farber 2017) were used to isolate *Vibrio* species from seafood samples. In brief, alkaline peptone water (1% peptone water, 2% NaCl, pH 8.5) was inoculated with homogenized seafood samples, while selective agar media TCBS—thiosulfate citrate bile sucrose (Difco)and CHROMAgar *Vibrio* (Dalynn Biologicals) were used to identify presumptive *Vibrio* isolates which were stored at –80 °C until needed. We noted that inoculation of seafood homogenate in 1% peptone in water at pH 8.5 and absence of NaCl increased the isolation rate of *V. cholerae* from seafood.

### Design of the study

The study was designed, planned, and performed according to the recommendations of the Clinical Laboratory Standards Institute (CLSI) Evaluation Protocol (EP) guidelines EP-09c, “Method Comparison and Bias Estimation Using Patient Samples” (Wayne 2018). Identification of the *Vibrio* isolates by WGS was considered as the reference method. The identification of the *Vibrio* isolates by MALDI-TOF MS was considered as the comparative method. In total, 161 bacterial isolates were analyzed by both methods, with the following breakdown: *V. cholerae, n* = 33; *V. parahaemolyticus, n* = 102; *V.* spp., *n* = 23; and other enteropathogens, *Salmonella* and *E. coli, n* = 3. All isolates in this study were isolated from seafood. These food samples yielded several different *Vibrio* species including the species of interest to this study: *V. cholerae* and *V. parahaemolyticus*. The representation of these two *Vibrio* species was addressed by using food samples collected in the period between year 2009 and year 2023. All identified *V. cholerae* isolates were used in this study.

Isolates with identification scores equal or greater than 2.0 using MBT Compass v4.1 software (Bruker) were considered as correctly identified species. Plotting and statistical analysis were performed with GraphPad Prism 10. Significance was calculated using a two-sided Fisher exact test and specificity and sensitivity by the Wilson-Brown test, with *p* < 0.05 considered significant.

### Whole genome sequencing

For sequencing, stocks were streaked onto tryptic soy agar with 2% NaCl (TSA-2N) and incubated at 35 °C overnight. A single colony was resuspended in a 400 µL of 1 × Zymo DNA/RNA Shield (Cedarlane). DNA was extracted using the Zymo Quick-DNA HMW Magbead Kit (with RNAseA treatment) as per the manufacturer (Zymo Research Corp). Paired-end Illumina sequencing was performed using NexteraXT libraries run on a MiSeq instrument (2 × 300 bp cycles, v3 chemistry) according to the manufacturer (Illumina Inc.).

Illumina reads were processed using BBMap v38.26 (sourceforge.net/projects/bbmap) to remove adapters and filter low-quality reads (BBDuk) and correct errors (Tadpole). De novo assemblies were generated using SKESA v2.4.0 (Souvorov et al. 2018) and error corrected using Pilon v1.23 (Walker et al. 2014). Assemblies were analyzed with QUAST v5.0.2 (github.com/ablab/quast) and taxonomically assigned using Mash v2.2.1 (Mash Screen (Ondov et al. 2016)) and the highest RefSeq (v93, ncbi.nlm.nih.gov/refseq) identities of > 0.8 (excluding hits containing the words phage or plasmid). Analysis tools were used with default settings unless noted. Details and accession numbers of isolates used in this study are shown in Table 1 (Table 1: List of isolates identified by WGS, MALDI-TOF, or biochemical tests and ID scores.).

**Table 1:**
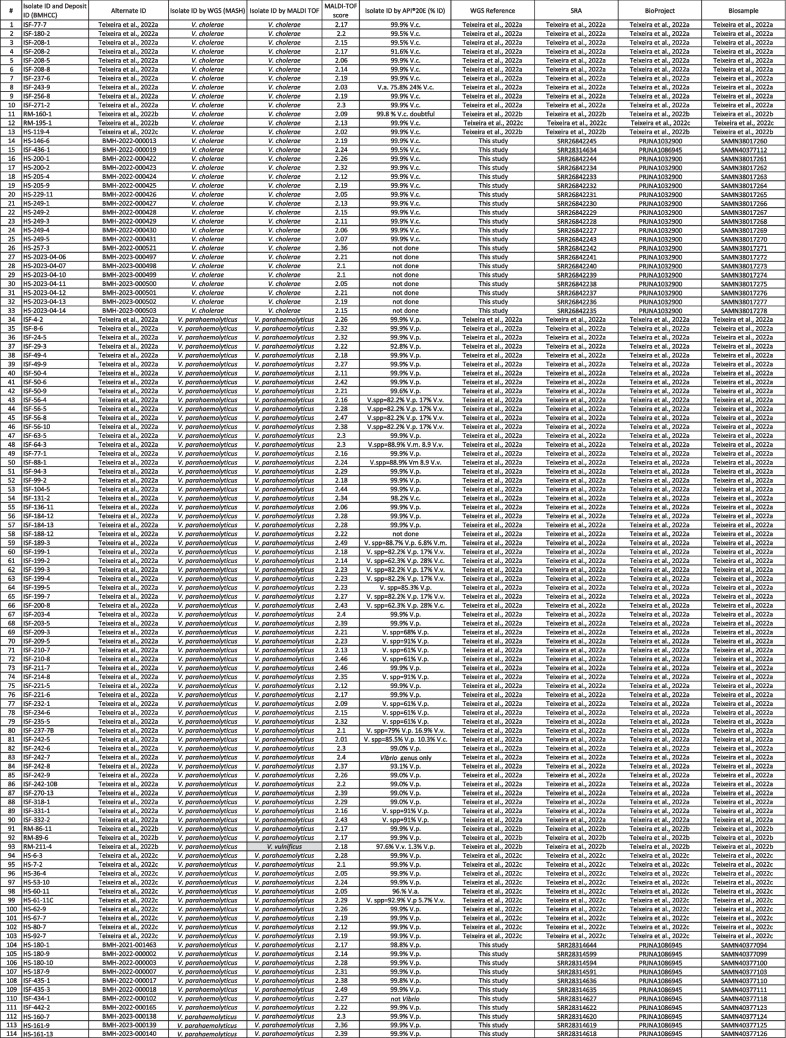

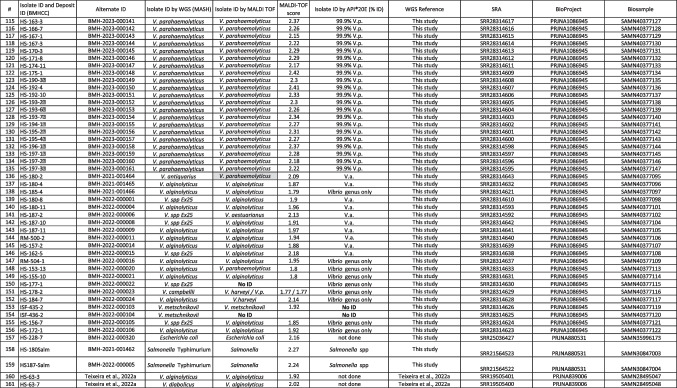
List of isolates identified by WGS, MALDI-TOF, or biochemical tests and ID scores

Note 1: *BMHCC*, Bureau of Microbial Hazards Culture Collection (https://ccinfo.wdcm.org/details?regnum=1306). Note 2: *V.* = Vibrio; *V.a.* = *V. alginolyticus*; *V.p.* = *V. parahaemolyticus*; *V.v.* = *V. vulnificus*; *V.c.* = *V. cholerae*; *spp* = species

Some isolates used in this study were described in previous reports (Teixeira et al. 2022a, 2022b, 2022c).

### Identification by biochemical methods

For the biochemical characterization of the strains in order to achieve a presumptive identification and not a definitive identification the analytical profile indexing, API®20E, test from Biomerieux (Cat. number 20160) was used as per manufacturer instruction. API®20E includes 20 different biochemical tests per isolate.

### Identification of *Vibrio cholerae* and *Vibrio parahaemolyticus* by MALDI-TOF MS

Preparation of *Vibrio* isolates for MALDI-TOF MS identification was performed according to the manufacturer’s instruction on the Biotyper RUO system based on microflex LT (Bruker Daltonik). Presumptive *Vibrio* species of interest that were stored at − 80 °C were resuscitated by streaking fresh colonies onto tryptic soy agar with 2% NaCl and incubated at 35 °C overnight. A single well-formed colony was chosen for Bruker’s standard operating procedure on extraction, version 2015. A single colony was collected with a sterile inoculating loop and then transferred to 1.5-mL tube containing 300 µL distilled water and resuspended; 900 µL anhydrous ethanol was added and mixed thoroughly. The suspension was centrifuged at 15,000 rpm (21,000 g) for 2 min and supernatant decanted. To remove residual ethanol, the tube was centrifuged again at 15,000 rpm for 2 min, and the pellet was dried at room temperature (RT) for 5 min. The pellet was then re-suspended in 30 µL of 70% formic acid (Honeywell Fluka). An equal volume of acetonitrile (Sigma-Aldrich) was added. Samples were mixed and subsequently centrifuged at 15,000 rpm for 2 min. Supernatant (1 µL) was transferred to a polished steel MSP 96 target plate (Bruker Daltonik) and allowed to dry at RT before being overlaid with 1 µL of matrix solution (HCCA—α-Cyano-4-hydroxycinnamic acid) Bruker Daltonik). The spectra were acquired within an hour after sample preparation on microflex LT instrument with flexControl v3.4 software and compared to the Bruker library of spectra, BDAL v11(10833). Spectra of *Vibrio cholerae* ATCC14033 were added to an internal library as per manufacturer instructions for identification of *V. cholerae* isolates.

The Bruker bacterial test standard (Bruker Daltonik) was used for calibration according to the manufacturer guideline. Identification scores of equal or greater than 2.0 indicated species-level identification, scores of 1.700–1.999 indicated genus-level identification, and scores of < 1.700 were considered unreliable. For this study, isolates with score equal or greater than 2.0 were considered as correctly identified species by the Bruker Biotyper.

## Results

### Performance evaluation of the MALDI-TOF MS method for identification of *Vibrio cholerae* and *Vibrio parahaemolyticus* isolates

#### *Vibrio cholerae*

**Fig. 1:**
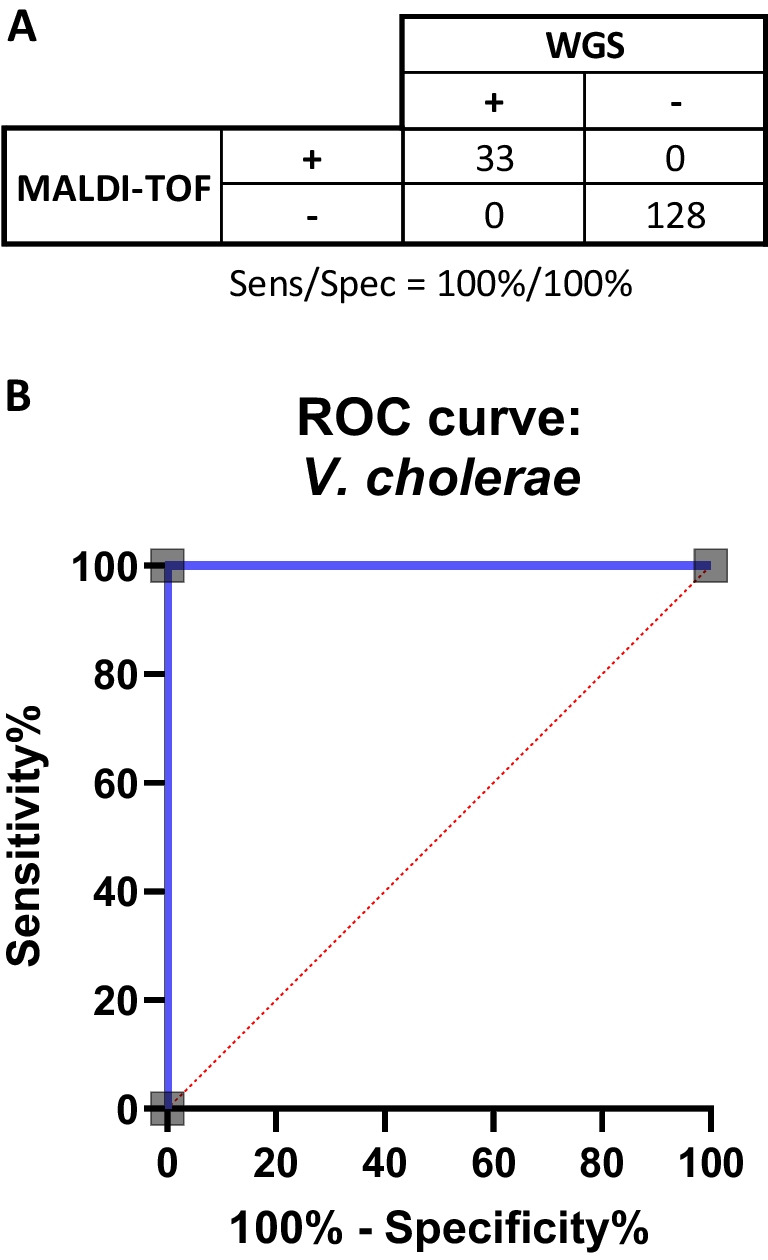
Performance evaluation of MALDI-TOF–MS in the identification of *V. cholerae*. **A** Sensitivity and specificity chart. **B** ROC curve

The MALDI-TOF MS system identified 100% (33/33) of the *V. cholerae* isolates, from total of 161 isolates tested. The statistical analysis indicates that the Bruker MALDI-TOF is a precise method for identification of *Vibrio cholerae* in comparison to the WGS method with high statistical significance *p* < 0.0001 per Fisher exact test. The results yielded a 100% sensitivity with a 95% CI of 0.8957 to 1 and 100% specificity with 95% CI of 0.9709 to 1.0 (Fig. 1A). The receiver operating characteristic—ROC yielded with a value of 1.0 AUC (area under the curve) (Fig. 1B).

#### *Vibrio parahaemolyticus*

**Fig. 2:**
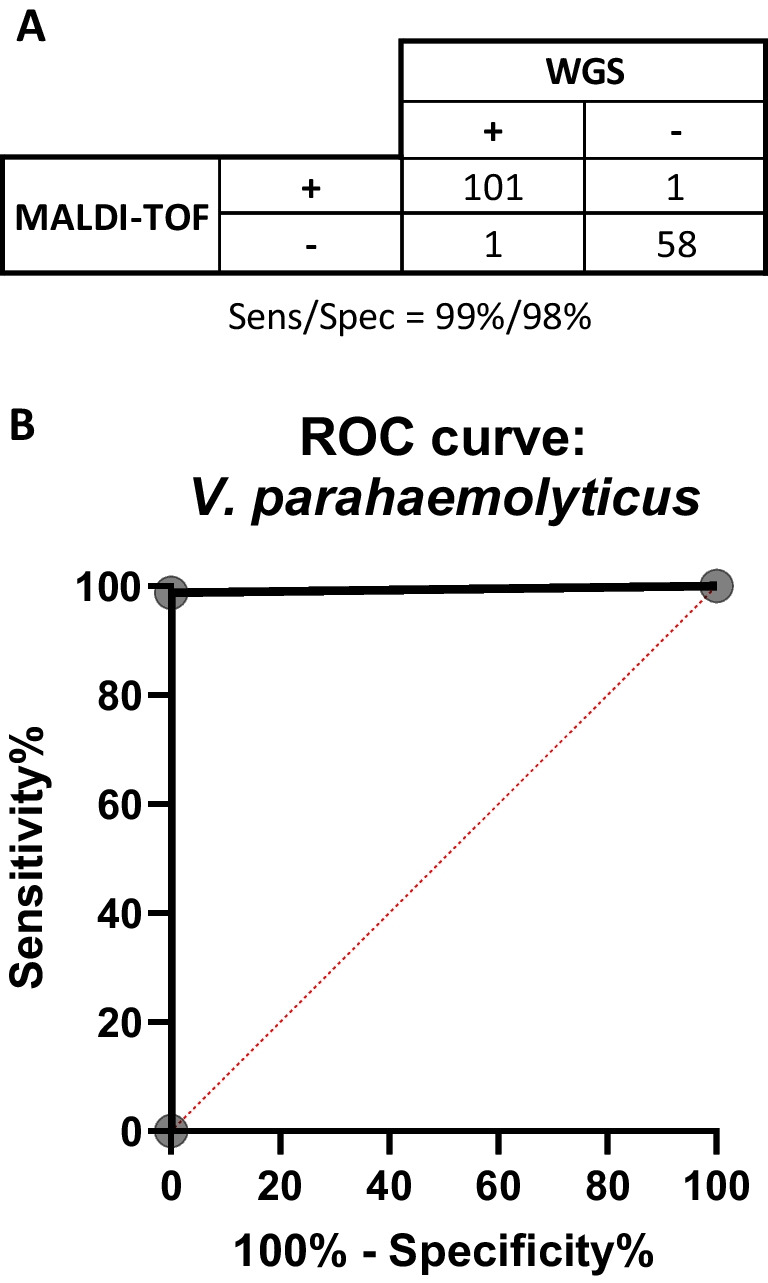
Performance evaluation of MALDI-TOF–MS in the identification of *V. parahaemolyticus*. **A** Sensitivity and specificity chart. **B** ROC curve

In the case of *V. parahaemolyticus*, the Bruker MALDI-TOF MS system identified 99.9% (101/102) *V. parahaemolyticus* isolates from a total of 161 isolates tested. The statistical analysis indicates that the Bruker MALDI-TOF MS is a precise method for identification of *Vibrio parahaemolyticus* in comparison to the WGS method with high statistical significance *p* < 0.0001 per Fisher exact test. The results yielded a 99% sensitivity with a 95% CI of 0.9465 to 0.9995 and 98% specificity with 95% CI of 0.91 to 0.9991 (Fig. 2A). The receiver operating characteristic yielded with a value of 0.9907 AUC (Fig. 2B).

From all of the tested isolates (*n* = 161), the MALDI-TOF MS method misidentified two isolates with a score higher than 2.0. An isolate identified as *V. parahaemolyticus* by WGS was identified by MALDI-TOF MS as a *V. vulnificus*, score 2.18 (a false negative result), and a *V. antiquarius* was identified by MALDI-TOF MS as a *V. parahaemolyticus*, score 2.09 (a false positive result), see Table 1.

## Discussion

The culture-dependent methods for biochemical characterization currently used in routine surveillance programs, which involve well-established techniques such as biochemical identification systems, are time consuming. These biochemical methods have been used extensively for the classification of *Vibrio* spp.; however, the accuracy of commercially available test systems is limited due to the high phenotypic diversity of *Vibrio* spp.

Efficiency, reliability, speed, and cost effective are the desired attributes of methods for identification of microorganisms in food matrices. The present-day methods such as the MALDI-TOF MS systems deliver those key attributes. However, the original study where the MALDI-TOF MS efficiency was tested in a multisite clinical study did not incorporate *Vibrio* isolates (Faron et al. 2015). Nonetheless, since then, other studies have been performed on the identification *Vibrio* spp., other than *V. cholerae* and *V. parahaemolyticus*, by MALDI-TOF alone on previously characterized isolates (Moussa et al. 2021) or use a combination of MALDI-TOF and sequencing of key genes (Dieckmann et al. 2010) or use of multi-locus sequence tagging for identification of *Vibrio* species (Jesser et al. 2019).

In this study, the sensitivity and specificity of Bruker’s MALDI-TOF MS Biotyper as a standalone method was evaluated in comparison to whole genome sequencing, with focus on identification of *Vibrio cholerae* and *Vibrio parahaemolyticus* food isolates. Identification of *V. cholerae* and *V. parahaemolyticus* by MALDI-TOF MS displayed an overall excellent conformity to the identification by whole genome sequencing. However, the low number of *V. cholerae* isolates used in the study is a technical limitation of the samples received, i.e., all *V. cholerae* isolated from seafood samples 2009–2023 are reported in this study. Ideally, we would prefer to have a higher number of *V. cholerae* isolates to increase the sample size, but that remains to be validated in future studies. The statistics comparing these two different methods in their conformity yielded high significance, underlying the necessity that adopting new technologies underscores the potential for increased productivity and efficiency. MALDI-TOF offers remarkably rapid, high throughput bacterial identification, but it does have challenges discerning closely related bacteria, e.g., BDAL v11(10,833) cannot distinguish between *V. vulnificu*s and *V. navarrensis*. While the technology itself confers certain limitations, improved resolution can be achieved by expanding the database to include underrepresented species, including *Vibrio* spp. (Liu et al. 2022; Mougin et al. 2020; Moussa et al. 2021). MALDI-TOF–based methods for detection and identification of *V. parahaemolyticus* and *V. cholerae* are clearly beneficial and have potential for use with other *Vibrio* and other bacteria relevant to food safety. Improved MALDI-TOF specificity of *V. parahaemolyticus* and *V. cholerae* identification for surveillance enables early public health interventions to mitigate health risk.

## Data Availability

All data supporting the findings of this study are available within the paper. Reads and assemblies have been deposited in NCBI under the accession numbers in Table 1 (BioProjects: PRJNA1086945, PRJNA1032900, and PRJNA880531). All isolates used in this study are deposited in BMHCC (Bureau of Microbial Hazards Culture Collection) WDCM1306 (World Data Centre for Microorganisms 1306)—https://ccinfo.wdcm.org/.
